# Correlation between knowledge of HIV, attitudes and perceptions of HIV and a willingness to test for HIV at a regional hospital in KwaZulu-Natal, South Africa

**DOI:** 10.4102/phcfm.v4i1.376

**Published:** 2012-07-18

**Authors:** Emeka E. Orisakwe, Andrew J. Ross, Peter O. Ocholla

**Affiliations:** 1 Department of Family Medicine, University of KwaZulu-Natal, South Africa; 2 Department of Earth Science-Geology, Stellenbosch University, South Africa

## Abstract

**Background:**

With millions of South Africans infected with human immunodeficiency virus (HIV) and less than 10% of the population aware of their HIV status, HIV counselling and testing (HCT) is the first step in any attempt to reduce the number of new infections. For those who test negative, HCT personalises the risks and reinforces preventative messages whilst for those who are positive, it is the gateway to accessing counselling and care. The Health Belief Model postulates that knowledge and attitude influence behaviour. The aim of this study was to determine whether knowledge of HIV and the attitude of patients referred for HCT correlated with a willingness to test for HIV.

**Methods:**

One hundred and seventy two patients referred for HCT were randomly selected over a three month period. Data were collected by a research assistant using the modified standardised World Health Organization (WHO)–Global AIDS Project (GAP) questionnaire.

**Results:**

Ninety per cent of the participants demonstrated sound knowledge of HIV, acquired immune deficiency syndrome (AIDS) and HCT. Despite the 90% of the participants with sound knowledge only 71.5% of the participants tested for HIV. There was no statistically significant difference in knowledge between those who tested and those who did not test for HIV. Twenty five per cent of those who refused to test stated that they had already made up their mind not to test for HIV before the counselling session.

**Conclusions:**

Despite excellent knowledge of HIV, a significant number of patients referred for HCT do not test for HIV.

## Abstrait

**Corrélation entre connaissances sur le VIH, attitudes et perceptions du VIH et volonté à passer un test de dépistage du VIH dans un hôpital régional au KwaZulu-Natal, en Afrique du Sud**

**Contexte:**

Des millions de Sud-africains étant infectés par le virus d'immunodéficience acquise (VIH) et moins de 10% de la population étant informés de son état sérologique, les conseils et le dépistage du VIH (CDV) constituent le premier pas vers une réduction du nombre de nouvelles infections. Pour les personnes dont le test est négatif, les CDV personnalisent le risque et renforcent les messages de prévention, tandis que pour celles dont le test est positif, ces CDV constituent une porte d'entrée vers une assistance socio-psychologique et une prise en charge. Le Modèle des croyances relatives à la santé établit le postulat selon lequel les connaissances et attitudes influencent le comportement. L'objectif de cette étude était de déterminer si les connaissances sur le VIH et l'attitude des patients transférés afin de bénéficier de CDV étaient corrélés à la volonté de passer un test de dépistage du VIH.

**Méthodes:**

Cent soixante-douze patients transférés afin de bénéficier de CDV ont été sélectionnés de manière aléatoire sur une période de trois mois. Les données ont été recueillies par un assistant chercheur en utilisant le questionnaire modifié standardisé de l'Organisation mondiale de la Santé (OMS) Global AIDS Project (GAP).

**Résultats:**

Quatre-vingt-dix pour cent des participants disposaient de solides connaissances sur le VIH, le syndrome d'immunodéficience acquise (Sida) et les CDV. En dépit du fait que 90% des participants disposaient de solides connaissances, seulement 71,5% des participants avaient passé un test de dépistage du VIH. Aucune différence statistiquement significative n'a été observée en termes de connaissances entre les personnes qui avaient passé un test de dépistage du VIH et celles qui n'avaient pas été testée. Vingt-cinq pour cent des personnes qui n'ont pas consenti a testeront déclaré avoir déjà pris la décision de ne pas prendre le test de dépistage VIH avant la séance d'assistance socio-psychologique.

**Conclusions:**

En dépit d'excellentes connaissances sur le VIH, un nombre significatif des patients transférés afin de bénéficier de CDV ne passent pas de test de dépistage du VIH.

## Introduction

### Key focus

Opinions vary amongst the general public, healthcare workers and the government regarding which of the two approaches is the more appropriate strategy to adopt towards the early detection of human immunodeficiency virus (HIV), that is, making use of ‘universal’ testing or ‘voluntary’ testing. The ethical, legal and health implications of either of these strategies could have far reaching implications for individuals in particular, and to the health system and society as a whole. Universal HIV testing will eventually result in everyone being tested provided that they come into contact with the public healthcare system. This will mean that we will be able to identify those individuals who are infected with the disease and, more importantly, we will be able to initiate treatment timeously as highly active anti-retroviral therapy (HAART) has been noted to improve morbidity, reduce mortality of infected patients. The ethical dilemma is that it runs the risk of violating the autonomy of an individual to self determination. On the other hand, voluntary HIV testing, which respects the individual's decision towards testing, reduces the chances of disease detection because many patients refuse to be tested and their autonomy has to be respected. As a result, these individuals could compromise their health as well as the health of others, as their unborn babies and their sexual partners are at risk of getting infected.

In South Africa, voluntary counselling and testing (VCT) was adopted in line with the World Health Organization (WHO) criteria which stipulates that it is necessary to respect an individual's autonomy and confidentiality in the management of the disease.

#### Background

HIV transmission, HIV infection and AIDS continue to be a massive challenge in South Africa. In July 2008, UNAIDS–WHO estimated that the prevalence of HIV amongst South African individuals aged between 15 and 49 years was 18.1%.^1^ This equates to 5.7 million South Africans living with HIV, including 280 000 individuals under the age of 15 years.^1^ The 2010 antenatal prevalence survey estimated that 40% of pregnant women in KwaZulu-Natal were HIV positive.^1^ Linked to the increasing prevalence of HIV there has been a dramatic rise in mortality rates in South Africa from 1997 when an estimated 316 559 people died of AIDS, to 2006 when 607 184 people died of an AIDS-related illness.^2^ In the absence of any vaccine to prevent HIV infection and antiretroviral therapy only being available in the public sector in SA since 2004, behaviour change has been seen as an important means of reducing the HIV incidence in SA. Millions of rands have been spent on intensive educational efforts by organisations such as Love Life, the Treatment Action Campaign (TAC), Khomanani, Brother for Life, and the South African government's drive to educate the country's population about HIV and to influence sexual behaviour. Despite these initiatives the prevalence of HIV continues to rise throughout the country.^2^

All of the HIV prevention programmes are based on the need for people to change their behaviour. Information is provided on issues related to HIV, its transmission, presentation, treatment options, preventative strategies, the right to say ‘no’, no discrimination, and access to treatment. The Health Belief Model postulates that knowledge, attitudes and perceptions of risk affect behaviour.^3^ Other behaviour change models, such as Social Cognitive Theory, emphasises that behaviour change is affected by environmental influences, personal factors, and attributes of the behaviour itself.^4^ With respect to HIV and/or AIDS these models have been used to gain a better understanding of human behaviour and factors that put people at risk as well as factors encouraging behavioural changes; if implemented these behavioural changes could reduce the morbidity and mortality associated with HIV related illnesses.^3^ In 2000 the Behavior Surveillance Surveys (BSS) published a large pool of data from several countries and continents showing that behavioural change can and does reduce risky sexual practices and drug addiction.^5^ The data gathered have been used by UNAIDS to highlight HIV prevention successes in countries such as Canada, India and Senegal, and to justify the behaviour change strategies.^5^

Voluntary counselling and testing (VCT) has been widely practiced in South Africa since 1996. The emphasis of VCT has been on the individual's autonomy and right to confidentiality. However, since the inception of VCT only a small percentage of South Africans have checked their HIV status.^2^ In response to the rising prevalence of HIV, and the perceived ineffectiveness of VCT, the South African government, in 2010, launched a massive HIV counselling and testing (HCT) campaign where counselling and testing was initiated by healthcare workers. The campaign was aimed at reducing by 50% the incidence of HIV by June 2011.^2^ The importance of knowing one's status through counselling and testing is recognised as a key strategy in fighting the HIV epidemic and is reflected in the National Strategic Plan (NSP) which has set targets of 70% of all adults in South Africa knowing their status by the end of 2011, and 25% of all adults having been tested for HIV in the last 12 months.^6^ The advantages of HCT and knowledge of one's status enable HIV negative patients to personalise their risks and reduce potentially risky behaviour. Those who test positive can begin ‘positive living’ and can access antiretroviral therapy. The National Department of Health reported in 2011 that in 2010 survey, 12 million South Africans were tested for HIV.^6^

Ngwelezana Hospital is a 554 bed secondary hospital complex situated in a semi-urban township area 180km north of Durban. The hospital serves an estimated population of 3 million people most of whom are unemployed Black Africans who reside in the adjacent rural and semi-urban areas.^7^ It is the referral hospital for 22 peripheral hospitals and 26 clinics. Each day about 4000 outpatients are seen and an average of 60 patients are admitted.^7^ Of these admissions, 60% – 70% are due to HIV related conditions, many of which are at an advanced stage of the disease.^7^ Prior to the introduction of the HCT programme, about 400 patients were referred for VCT each month. Since the advent of the HCT programme, about 900 patients have presented for HCT each month.^7^ Most patients are referred for HCT by healthcare workers because of opportunistic infections suggestive of HIV infection, prior to circumcision and when pregnant, with only a small number of patients being self-referred.^7^ Considering the massive drive for HIV counselling and testing as well as the ongoing educational initiatives in the mass media, one would expect that all the patients referred for HCT would consent to testing. However, reports from the counsellors suggest that only 75% – 95% of patients referred for HCT consent to getting tested.^7^

#### Objectives of the study

There is limited data on why patients test or refuse to test for HIV at Ngwelezana Hospital. The aim of this study was to determine whether knowledge of HIV and the attitude of patients referred for HCT correlated with a willingness to test for HIV. Feedback from the study will be provided to the counselling service to strengthen the HCT programme.

#### Significance of the study

This study highlights the reasons that create difficulties in achieving a 100% HIV testing rate despite the availability of testing facilities as well as the ongoing campaigns which have resulted in improved knowledge about the disease. These difficulties contextualise the notable Health Belief Model Theory which is based on the psychological model that attempts to explain and predict health behaviours by focusing on the attitudes and beliefs of individuals.

## Methods

### Material, setting and design

The study was a cross-sectional observational descriptive study. A sample size of 172 patients was selected, in consultation with the biostatistian, on the assumption that 75% of the participants will consent to HIV testing to achieve 80% power of study which will detect a difference. Counsellors at the HCT clinic provide pre-test counselling, conduct the rapid HIV test and then provide appropriate post-test counselling depending upon the result of the test. As patients present at the HCT clinic in a random order, the research assistant selected every third patient in the queue who was 18 years of age and over, until the required number was reached. All patients had been referred for HCT during the study period, which was between October 2010 and December 2010. If the selected patient declined to participate, the next person was chosen. Permission to conduct the study was obtained from the Post Graduate and Bio Ethics committee of the University of KwaZulu-Natal, the Department of Health in KwaZulu-Natal and Hospital Management. Written consent was obtained from all the patients before they completed the questionnaire and all patients were assured of the confidentiality of the information.

### Procedure and analysing

Data were collected using a structured questionnaire adapted from a modified, standardised WHO–GAP questionnaire.^8^ The questionnaire was administered in two parts: pre-counselling and post-counselling. Whilst patients were waiting in the queue the pre-counselling section of the questionnaire was administered which collected data on the demographic profile, knowledge of HIV, attitudes towards HIV and participants’ perceptions of risk. The post-counselling section was administered when participants exited from the counsellor's office and data were then collected in terms of whether or not they had an HIV test and the reasons for their choice. Whether or not the patient tested for HIV was confirmed with the counsellor after the questionnaire was completed. Data were entered in SPSS and further analysis involved descriptive statistics, cross tabulations, pair wise multiple comparisons, Post Hoc test and, ANOVA statistics. A *p*-value of < 0.05 was considered significant.

## Ethical consideration

Informed consent was obtained from all participants; some exclusion criteria were also observed, such as: any attendee who has already tested for HIV, any patient who is mentally handicapped or critically ill, any patient who refuses to consent to the study, and under-aged patients. The University of KwaZulu-Natal Biomedical Research Ethics Committee approval (BE 151/09) was obtained before the commencement of this study.

## Results

One hundred and seventy two (172) participants completed the questionnaire. All patients approached to participate in the study, agreed to take part. The oldest participant was 74 years old. The majority of patients were referred from peripheral clinics with only nine patients being self-referred. Most patients were single (74.4%), 56.9% had completed secondary education, and 18.6% had tertiary qualifications. One hundred and thirteen (65.7%) of the participants were unemployed and six participants (3.5%) were self-employed (Table 1).

**TABLE 1:** Socio-demographic information of the participants.

Gender	Male		Female		Total	
		
	*n*	% = 50.6	*n*	% = 49.4	*N* = 172	%
**Age distribution**						
Less than 20 years	7	4.1	4	2.3	11	6.4
20–24	21	12.0	15	9.0	36	21.0
25–29	16	9.3	19	11.0	35	20.3
30–34	11	6.4	10	5.8	21	12.2
35–39	4	2.3	11	6.4	15	8.7
40 and above	28	16.3	26	15.1	54	31.3
**Level of education**						
None	8	4.7	3	1.7	11	6.4
Informal	5	2.9	11	6.4	16	9.3
Primary	6	3.5	9	5.2	15	8.7
Secondary	49	28.5	49	28.5	98	57.0
Tertiary	19	11.0	13	7.6	32	18.6
**Source of referral**						
Clinic	70	41.0	64	37.0	134	78.0
Hospital	6	3.5	9	5.2	15	8.7
General Practitioner	6	3.5	8	4.6	14	8.1
Self	5	2.9	4	2.3	9	5.2
**Race**						
African	84	48.8	74	43.0	158	91.8
Caucasian	1	0.6	5	2.9	6	3.5
Indian	2	1.2	4	2.3	6	3.5
Coloured	0	0	2	1.2	2	1.2
**Marital status**						
Married	14	8.1	20	11.6	34	19.7
Single	67	38.9	61	35.4	128	74.3
Widowed	5	2.9	4	2.3	9	5.2
Cohabiting	1	0.6	0	0	1	0.6
**Employment**						
Not employed	59	34.3	54	31.4	113	65.7
Formal employment	18	10.5	15	8.7	33	19.2
Self employed	6	3.5	10	5.8	16	9.3
No response	4	2.3	6	3.4	10	5.7

*n*, Given as number of participants.

Of the 172 participants, only 123 (71.5%) tested for HIV of whom 41 (33%) were HIV positive. Thirty of those who were tested for HIV informed the research assistant that they had not previously been tested. Of these 30 participants, 70% (22) were HIV positive. There was no significant difference in testing between gender, level of education, employment or age (Table 2).

**TABLE 2:** Socio-demographics and human immunodeficiency virus testing of the participants.

Demographics	Testing: Yes		Testing: No		Total		*p*-value
			
	*n = 123*	% = 71.5	*n = 49*	% = 28.5	*N* = 172	% = 100	
**Gender**							0.35
Male	65	38.0	22	13.0	87	51.0	-
Female	58	33.0	27	16.0	85	49.0	-
**Level of education**							0.47
No form of education	9	5.2	2	1.2	11	6.4	-
Informal education	10	5.8	6	3.5	16	9.3	-
Primary education	12	7.0	3	1.7	15	8.7	-
Secondary education	73	42.0	25	15.0	98	57	-
Tertiary education	19	11.0	13	7.6	32	18.6	-
**Employment**							0.40
Employed	24	14.0	9	5.2	33	19.2	-
Unemployed	83	48.3	30	17.4	113	65.7	-
Self employed	10	5.8	6	3.5	16	9.3	-
No response	6	3.5	4	2.3	10	5.8	-
**Age category**							0.49
Less than 20 years	5	2.9	6	3.5	11	6.4	-
Between 20 and 24	26	15.0	10	6.0	36	21.0	-
Between 25 and 29	22	12.8	13	7.5	35	20.3	-
Between 30 and 34	14	8.1	7	4.1	21	12.2	-
Between 35 and 39	11	6.4	4	2.3	15	8.7	-
40 and above	45	26.2	9	5.2	54	31.4	-

*n*, Given as number of participants.

The most common reasons cited by participants for testing was the desire to know their HIV status and to better understand the HIV disease whilst the most common reason cited for *not* testing was that participants had already decided not to test (Table 3).

**TABLE 3:** Reasons for human immunodeficiency virus testing and reasons for refusing tests.

Reasons	Yes		No		Unsure		Total	
			
	*n*	%	*n*	%	*n*	%	*n*	%
**Testing encouragementby:**								
Boyfriend or girlfriend	14	8.1	73	42.4	85	49.4	172	100
Your spouse	18	10.5	66	38.4	88	51.2	-	-
The health worker	33	19.2	53	30.8	86	50.0	-	-
The counsellor	38	22.1	49	28.5	85	49.4	-	-
Your co-worker	9	5.2	73	42.4	90	52.3	-	-
Media campaigns on HIV infection	27	15.7	52	30.2	93	54.1	-	-
Your choice to know your status	81	47.1	10	5.8	81	47.1	-	-
A better understanding of HIV	71	41.3	16	9.3	85	49.4	-	-
Knowledge of treatment	55	32.0	27	15.7	90	52.3	-	-
**Declining test encouragement by:**								
Boyfriend or girlfriend	5	2.9	32	18.6	135	78.5	-	-
Your spouse	7	4.1	37	21.5	128	74.4	-	-
The health worker	6	3.5	38	22.1	128	74.4	-	-
The counsellor	7	4.1	37	21.5	128	74.4	-	-
Your co-worker	5	2.9	39	22.7	128	74.4	-	-
Made up your mind not to test	44	25.6	23	13.4	105	61.0	-	-
Don't want to know your status	22	12.8	29	16.9	121	70.3	-	-

*n*, Given as number of participants.

HIV, Human immunodeficiency virus.

According to the patients who participated in the study, information about HIV was obtained from many sources including Television, radio, family members and the clinic. The clinic was reported as being the major source of information on VCT (92.4%) and HIV (90.7%) (Table 4). Generally across the educational spectrum, participants exhibited excellent knowledge about HIV, its mode of transmission and preventative measures (Table 5). Abstinence, faithfulness and condom use were correctly identified as preventative measures by most of the participants. However, 13% of the sample reported that taking a shower after sex is a preventative measure (Table 5).

**TABLE 4:** Awareness and source of information about voluntary counselling and testing and human immunodeficiency virus.

Source	Clinic		Radio		Television		Newspaper		Relative or friend		Partner	
					
	*n*	%	*n*	%	*n*	%	*n*	%	*n*	%	*n*	%
**Voluntary counselling and testing information Response**												
Yes	159	92.4	153	89.0	147	85.5	137	79.7	126	73.3	120	69.8
No	8	4.7	7	4.1	10	5.8	14	8.1	25	14.5	25	14.5
Unsure	5	2.9	12	7.0	15	8.7	21	12.2	21	12.2	27	15.7
**HIV Information**												
Yes	156	90.7	135	78.3	136	74.1	127	73.8	117	68.0	107	62.2
No	0	-	4	2.3	5	2.9	6	3.5	13	7.6	17	9.9
Unsure	16	9.3	33	19.2	31	22.7	39	22.7	42	24.4	48	27.9

*n*, Given as number of participants.

HIV, Human immunodeficiency virus.

**TABLE 5:** Level of education and knowledge of human immunodeficiency virus.

Correct knowledge of	Cross tabulation of level of education and affirmative responses to the options										*P*-value

	No form of education		Informal		Primary		Secondary		Tertiary		
				
	*n* = 11	%	*n* = 16	%	*n* = 15	%	*n* = 98	%	*n* = 32	%	
**HIV transmission**											
Sexual transmission	10/11	91	16/16	100	13/15	87	92/98	94	29/32	91	0.915
Mother-to-child	8/11	73	14/16	88	12/15	80	87/98	89	26/32	81	0.966
Blood transfusion	10/11	91	15/16	100	11/15	73	92/98	94	28/32	88	0.048
Blood on skin cut	10/11	91	16/16	100	12/15	80	89/98	91	28/32	88	0.418
Sharing needles	10/11	91	14/16	88	10/15	67	67/98	68	26/32	81	0.357
Kissing	3/11	27	3/16	19	6/15	40	21/98	21	9/32	28	0.439
**HIV prevention**											
Abstinence	10/91	91	14/16	88	14/15	93	89/98	91	29/32	91	0.980
Faithful to partner	10/11	91	15/16	94	14/15	93	86/98	88	30/32	94	0.954
Multiple sexual partners	6/11	55	8/16	50	4/15	27	19/98	19	6/32	19	0.027
Polygamy	4/11	36	4/16	25	2/15	13	15/98	15	6/32	19	0.407
Condom use	8/11	73	14/16	88	14/15	93	88/98	90	31/32	97	0.089
Shower after sex	2/11	18	4/16	25	2/15	13	17/98	17	2/32	6	0.244
ART prophylaxis	4/11	36	7/16	44	6/15	40	38/98	39	15/32	47	0.424
There is a link between HIV and tuberculosis	6/11	55	15/16	94	13/15	87	84/98	86	25/32	78	0.289

*n*, Given as number of participants.

HIV, Human immunodeficiency virus; ART, anti-retroviral therapy.

The majority of the participants across all levels of education had excellent knowledge about the relationship between HIV and Tuberculosis (TB). Participants with higher levels of education did not demonstrate better knowledge when compared to those with less education (Table 5).

In terms of attitudes towards HIV, 159 participants (92.4%) indicated that they considered those infected with HIV to have brought it upon themselves (Table 6). Despite this, 88 participants (51.2%) indicated that they would show support to those individuals living with HIV.

**TABLE 6:** Attitude and perceptions towards human immunodeficiency virus and the practice of voluntary counselling and testing.

Variables	Agree		Disagree		Unsure		*p-*value
		
	*n*	%	*n*	%	*n*	%	
**Attitude towards HIV infection**							
Individuals infected with HIV have brought it upon themselves.	159	92.4	5	2.9	8	4.7	0.305
I will show support to those living with HIV.	88	51.2	46	26.7	38	22.1	0.244
**Severity of HIV disease**							
HIV is a serious disease.	158	91.9	4	2.3	10	5.9	0.121
**Susceptibility to HIV disease**							
Young people are not at risk of HIV.	46	26.7	110	64.0	16	9.3	0.733
Having a baby predisposes one to HIV.	104	60.5	43	25.0	25	14.6	0.829
Drugs and/or alcohol put one at risk of HIV.	128	74.4	25	14.5	19	11.0	0.077
Being a man reduces the risk for HIV.	55	32.0	89	51.7	28	16.3	0.169
You are at risk for HIV.	105	61.8	36	21.2	29	17.0	0.017
**The following are barriers to HIV testing**							
Discrimination by employer.	61	35.7	59	34.5	51	29.9	0.005
Discrimination by insurance companies.	70	40.9	42	24.6	59	34.5	0.014
Discrimination by Department of Health because anti-retroviral therapies are only initiated at a CD4 count of 200 or less.	70	40.9	46	26.9	54	32.1	0.660
**Benefits of HIV testing**							
Knowing one's HIV status.	163	95.3	2	1.2	6	3.5	0.140
To seek early medical help.	163	94.8	1	0.6	8	4.6	0.380

*n*, Given as number of participants.

HIV, Human immunodeficiency virus.

One hundred and five participants (61%) felt that they might be at risk of being HIV positive (Table 6). Drugs and alcohol, risky sexual behaviour and the desire to have a baby were perceived by participants as high risk factors associated with infection (Table 6). Barriers identified in terms of HIV testing included discrimination by insurance companies if the test result is positive (70, 40.9%), unavailability of anti-retroviral therapy for those with a CD4 count of more than 200, and participants’ concern about discrimination by employers (Table 6).

## Discussion

The demographics of this study reflect the population from which the study population was drawn: participants were Black, young and unemployed.

This study has shown that knowledge of HIV, its transmission and appropriate protective measures are excellent across all age groups, both genders and all education levels. In 2005, Nachega et al. reported high levels of knowledge about HIV in Soweto with no association between education, gender, age and extent of knowledge.^9^ It is encouraging to note that health education, provided through mass media (i.e. radio and television), and clinics are associated with a high level of knowledge of HIV. This is consistent with international studies which have shown that television and radio are effective means of informing the population about HIV and AIDS.^10, 11^ The finding in this research, that those who had a secondary education tested more frequently than those in other groups, is consistent with the SA UNAIDS report of 2009, which showed a positive correlation between HIV testing and education.^2^ It was surprising, therefore, that this trend did not persist with those who had a tertiary education. Despite this a number of myths persist, most notably, the myth made famous by the president of South Africa, that taking a shower after sex reduces the risk of HIV.^12^ It is important that these myths are challenged and educational initiatives introduced to address erroneous information such as this.

It was also encouraging to note that the majority of participants view HIV as a serious disease. However, it is of concern to note that there was no correlation between knowledge, attitudes, perceptions of risk and testing. Despite excellent knowledge and 94% of the patients identifying advantages to testing, only 123 (71.5%) of the study participants tested for HIV which is lower than the testing rate of 75% – 95% reported by the HCT counsellors.^7^ This is not a new finding and is consistent with other studies. Kalichman et al. (2003) reported that amongst township youth in Cape Town, despite a high mean score of 83% in knowledge, there was no significant difference between those who knew their status and those who did not.^13^ This gap between knowledge and action is crucial to understanding and further research is needed in this area. These findings challenge the simplistic view of the Behavior Change Model which postulates that better knowledge leads to better action.

It is of concern that, of the patients interviewed, only 61% considered themselves to be at risk despite the fact that 91% of them were referred. The reasons for referral for HCT were not ascertained, but it would not be unreasonable to assume that this was a sexually active, high risk group, with whom the possibility of HIV infection would have been discussed prior to referral. This again highlights the gap between knowledge, personalising the risk and acting on that information. Our study indicated that half of the participants did not consider the male population or youth to be associated with less risk in contracting HIV. In studies conducted by Taylor et al. in 2002 and 2007, the majority of male high school learners and young people surveyed did not see themselves as susceptible to HIV despite the very high prevalence of HIV in South Africa.^14, 15^ The model of reasoned action and the theory of planned behaviour recognise that in addition to knowledge, perception of risk, self actualisation and community expectations influence behaviour. With only 61% of participants considering themselves to be at risk, more work needs to be done to help patients recognise and personalise risk.

The most common reason given for testing was a desire to know one's status. Media emphasis has highlighted the importance of knowing one's status with campaigns, such as the ‘HIV free generation – It begins with you’ campaign. Combined with the availability of ART, advocacy campaigns that focus on raising HIV awareness has broadened the public's understanding and perception of VCT.^16^

The most common reason for non-testing, in this study, was ‘already decided’. This decision means that certain participants had already made up their mind not to test despite waiting to consult the counsellor for their pre-counseling session. It is not clear from the study why the patients were referred for HCT if they had already decided not to test. It may be that the patients needed to hear more about HIV or to understand more about the disease and the advantages and disadvantages of testing. This occurrence, in terms of participants who had ‘already decided’ not to get tested for HIV, will need further investigation to enable counsellors to successfully address such patients in the future. A study done in Durban at the University of KwaZulu-Natal in 2006 indicated that perceived social stigma, personal fear and social support affected the willingness of first year students to test for HIV.^17^

Other factors mentioned by 40% of the sample as reasons for not getting tested included discrimination by insurance companies and the knowledge that the Department of Health would only initiate ART with a CD4 count of 200 or less. These findings are consistent with those of Day et al. (2003) which showed that amongst gold miners, fear of testing positive and subsequent stigmatisation were the major barriers to HIV testing.^18^ We were surprised that during the exit interview, 30 out of the 123 participants deliberately told the research assistant that they did *not* test for HIV despite having indeed tested after the pre-counselling session. Twenty-two (22) participants out of the 30 were HIV positive. This unwillingness to disclose their HIV status to the research assistant, despite confidentiality being assured, needs further study and may signify a fear of stigmatisation, denial or an unwillingness to disclose their status to their partners and close family members. It was concerning to note that less than 20% indicated that encouragement by a sexual partner was the reason for getting tested, suggesting that issues surrounding sex and/or sexuality are not commonly discussed in intimate relationships. This needs further investigation as HIV affects both sexual partners; a discussion of risk factors and preventative measures should take place between all sexually active individuals and their sexual partners. In a study in Soweto conducted in 2005, Nachega et al. reported that 38% of the 73 participants did not disclose their HIV status to their sexual partners.^9^ Nachega concluded that the common denominator in terms of non-disclosure was culturally-based gender differences in terms of personal power which results in a fear of one's partner's reactions and general attitude.^9^ These findings were also attributed to the traditional African culture which is patriarchal in nature and perceived to be oppressive to women, undermining their ability to negotiate safer sexual practices.^19^

### Limitations of the study

The direct nature of some of the questions may be a source of information bias. Cultural limitations may also introduce bias as some participants who are from rural areas still identify strongly with certain traditional beliefs which may not permit giving answers to some sexual questions. The majority of the population that uses the Ngwelezana hospital comprises African people which mean that the results are reflective of one race only. Future research in a setting that encompasses cultural and racial diversity with mixed socio-economic and educational backgrounds will be beneficial.

### Recommendations

Counsellors have to be trained to achieve skills useful in effective communication. The skills will help in the following areas:

- The non-opinionated interrogation of patients in an attempt to identify those participants who had ‘already decided’ not to test even before the pre-counselling sessions take place.
- The ability to help those identified with the above reason to verbalise the reasons why they have made up their mind not to test even before attending counselling sessions.
- To be able to provide quality targeted information, create confidence and reach an agreement with the patients, which will aid in an active re-decision to test for HIV or to return for further counselling.

## Conclusion

The majority of the participants demonstrated excellent knowledge about HIV and/or AIDS. However, despite this a significant number of patients referred for HCT did not recognise that they might be at risk of HIV and did not agree to test for HIV. There was no statistical correlation between age, gender, employment, level of education and willingness to test. A reluctance to disclose one's HIV status suggests a significant fear of discrimination. Despite the major emphasis on knowing ones status, more research is required to better understand the reasons for non-testing so that more effective education and counselling can be provided.
